# Finite-temperature gluon spectral functions from $N_f=2+1+1$ lattice QCD

**DOI:** 10.1140/epjc/s10052-018-5593-7

**Published:** 2018-02-14

**Authors:** Ernst-Michael Ilgenfritz, Jan M. Pawlowski, Alexander Rothkopf, Anton Trunin

**Affiliations:** 1 Bogoliubov Laboratory of Theoretical Physics, Joint Institute for Nuclear Research, Joliot-Curie Str. 6, 141980 Dubna, Russia; 2 Institut für Theoretische Physik, Universität Heidelberg, Philosophenweg 16, 69120 Heidelberg, Germany; 3 ExtreMe Matter Institute EMMI, GSI Helmholtzzentrum für Schwerionenforschung mbH, 64291 Darmstadt, Germany; 4 Institut für Theoretische Physik, Universität Heidelberg, Philosophenweg 12, 69120 Heidelberg, Germany

## Abstract

We investigate gluon correlation functions and spectral functions at finite temperature in Landau gauge on lattice QCD ensembles with $N_f=2+1+1$ dynamical twisted-mass quarks flavors, generated by the tmfT collaboration. They cover a temperature range from $0.8\le T/T_C\le 4$ using the fixed-scale approach. Our study of spectral properties is based on a novel Bayesian approach for the extraction of non-positive-definite spectral functions. For each binned spatial momentum we take into account the gluon correlation functions at all available discrete imaginary frequencies. Clear indications for the existence of a well defined quasi-particle peak are obtained. Due to a relatively small number of imaginary frequencies available, we focus on the momentum and temperature dependence of the position of this spectral feature. The corresponding dispersion relation reveals different in-medium masses for longitudinal and transversal gluons at high temperatures, qualitatively consistent with weak coupling expectations.

## Introduction

Understanding the evolution of strongly interacting matter in a heavy-ion collision is one of the most demanding tasks in current theoretical physics [1]. Not only do we need to describe the real-time dynamics of matter in the high-temperature phase of QCD, the quark–gluon plasma, but also its transition to the low-temperature domain of hadrons seen and measured in experiment. In particular around the chiral cross-over transition, estimated on the lattice [2–5] to occur at $T_c=155\pm 9$ MeV, it is vital to uncover how the breaking of chiral symmetry and the onset of confinement proceed and how they affect the relevant degrees of freedom in the system.

Transport and thermal properties of strongly interacting matter have been extracted from the correlation functions of mesons, i.e. hadronic observables. For computations based on lattice simulations see e.g. [6–13, 60]. On the other hand such real-time properties can also be accessed via the spectral functions of the fundamental constituents of QCD, gluons and quarks; see e.g. [14, 19–25].

In the present study we focus on the gluonic sector. The study of gluon correlation functions in gauge-fixed QCD has garnered interest for quite some time, both with lattice simulations and with functional approaches, for finite-temperature results see e.g. [26–31] and [32–39], respectively. The extraction of the corresponding spectral functions is, however, hampered by the fact that gauge-fixed gluon spectra contain non-positive-definite contributions; see e.g. [40]. This in turn defies standard approaches, based on Bayesian inference, such as the Maximum Entropy Method (MEM) [41]. In turn direct extensions [22, 42], modifications of the prior [20], as well as of the data part, such as the introduction of shift functions [21], have been applied in the literature. Over the last few years progress has been made in developing new Bayesian approaches independent from the MEM [43], which recently have also been generalized to non-positive-definite spectra [14]. Some non-Bayesian approaches, such as the Backus–Gilbert method [44] or the Sumudu transformation [45] also allow for the treatment of spectra with negative contributions.

Investigating the spectral properties of gluons serves several complementary purposes; first and foremost it provides a direct and intuitive handle on phenomena, such as the generation of a mass gap in the context of confinement [40, 46] as well as the emergence of thermal masses related to Debye screening.

Secondly gluon spectral functions play a vital role in the self-consistent computation of transport coefficients in functional approaches to QCD [19, 21], such as the functional renormalization group or Dyson–Schwinger approaches. If the gluon spectral function is known, it may serve as input to a closed set of real-time evolution equations for quark and gluon degrees of freedom, from which relevant quantities, such as energy-momentum correlation functions may be computed. Thus, in turn, transport properties become accessible.

From a practical point of view, we are also interested in using lattice gluon spectral functions to validate phenomenological models used in the description of heavy-ion collisions. Some of these rely on a quasi-particle picture for the fundamental constituents of strongly interacting matter, which at high temperature is matched to resummed perturbative predictions from hard-thermal loops. One example in this regard is the parton–hadron string dynamics model [47–49]. Elucidating the non-perturbative behavior of the gluon spectral function may therefore lead to more refined approximations and a better understanding of the validity of currently used model assumptions.

Single particle properties in QCD are conceptually more difficult to capture than those of e.g. mesons. Quarks and gluons represent color charged fields and thus their correlation functions are not gauge invariant. Hence gauge fixing becomes necessary and one has to carefully understand which of the observed properties is truly physical and which depends on the choice of gauge. Dismissing altogether the study of gauge dependent correlators, however, is a too narrow point of view, as they may still contain gauge independent information. One example is the extraction of the heavy-quark potential from Wilson-line correlators in Coulomb gauge [50–52]. In that case the gauge independent spectral feature encoding the potential is embedded in a gauge dependent background, which may be cleanly separated.

In this first study of gluon properties in thermal lattice QCD with $N_f=2+1+1$ dynamical twisted-mass flavors, we use the conventional choice of Landau gauge $\partial ^\mu A_\mu ^a=0$, which is manifestly Lorentz invariant and retains global $SU(N_c)$ gauge symmetry. In the following the computations are carried out in the Euclidean domain, where time has been Wick-rotated to $\tau =it$. The gluon correlator is defined from the Fourier transformed gauge fields $\widetilde{A}^a_{\mu }(q)$

1 $$\begin{aligned} D^{ab}_{\mu \nu }(q) =\left\langle \widetilde{A}^a_{\mu }(q)\widetilde{A}^b_{\nu }(-q) \right\rangle . \end{aligned}$$

In the presence of a thermal bath, Lorentz invariance and hence Euclidean rotational invariance is broken and one may split the correlator into a transversal $D_T$ (“chromomagnetic”) and a longitudinal $D_L$ (“chromoelectric”) component

2 $$\begin{aligned} D^{ab}_{\mu \nu }(q)=\delta ^{ab} \left( P^{T}_{\mu \nu } D_{T}(q_{4}^{2},\mathbf {q}^{\,2})+ P^{L}_{\mu \nu } D_{L}(q_{4}^{2},\mathbf {q}^{\,2}) \right) . \end{aligned}$$

For the particular choice of Landau gauge the projectors $P^{T,L}_{\mu \nu }$ are aligned transversally or longitudinally with the imaginary frequency $(\mu =4)$ direction:

3 $$\begin{aligned} P^{T}_{\mu \nu }= & {} (1-\delta _{\mu 4})(1-\delta _{\nu 4}) \left( \delta _{\mu \nu }- \frac{q_{\mu }q_{\nu }}{\mathbf {q}^{\;2}}\right) , \end{aligned}$$

4 $$\begin{aligned} P^{L}_{\mu \nu }= & {} \left( \delta _{\mu \nu }-\frac{q_{\mu }q_{\nu }}{q^2}\right) -P^{T}_{\mu \nu }\;. \end{aligned}$$

The explicit expressions for the propagators $D_{T,L}$ read

5 $$\begin{aligned} D_T(q)=\frac{1}{2 N_g} \left\langle \sum _{i=1}^3 \widetilde{A}^a_i(q) \widetilde{A}^a_i(-q) -\frac{q_4^2}{\mathbf {q}^{\;2}} \widetilde{A}^a_4(q)\widetilde{A}^a_4(-q) \right\rangle \end{aligned}$$

and

6 $$\begin{aligned} D_L(q)= \frac{1}{N_g}\left( 1 + \frac{q_4^2}{\mathbf {q}^{\;2}}\right) \left\langle \widetilde{A}^a_4(q) \widetilde{A}^a_4(-q) \right\rangle , \end{aligned}$$

with $N_g=N_c^2-1$ and $N_c=3$. This fixes also the dressing functions $Z_{T,L}(q)=q^2 D_{T,L}(q)$, which are often considered in functional computations. If we wish to evaluate the zero four-momentum correlator, we have to consider how to handle the ratios $\frac{q_4^2}{\mathbf {q}^{\;2}}$, in particular since at $T>0$ the limits $q_4\rightarrow 0$ and $|q|\rightarrow 0$ do not commute. Here we choose to let go $q_4\rightarrow 0$ first, as it provides access to the screening masses

7 $$\begin{aligned} D_T(0)= & {} \frac{1}{3 N_g} \sum _{i=1}^3 \left\langle \widetilde{A}^a_i(0) \widetilde{A}^a_i(0) \right\rangle , \end{aligned}$$

8 $$\begin{aligned} D_L(0)= & {} \frac{1}{N_g} \left\langle \widetilde{A}^a_4(0) \widetilde{A}^a_4(0) \right\rangle . \end{aligned}$$

Note that the factor 1 / 3 instead of 1 / 2 is related to the difference in the trace over the projectors for finite and vanishing $q_4$.

We may relate the gluon correlators in imaginary frequencies $q_4$ to their spectral function via the Källen–Lehmann representation

9 $$\begin{aligned} D_{T,L}(q_4,{\mathbf q})= & {} \int _{-\infty }^\infty \frac{1}{iq_4-\omega } \rho _{T,L}(\omega ,{\mathbf q}) \mathrm{d}\omega \nonumber \\= & {} \int _0^\infty \frac{2\omega }{q_4^2+\omega ^2} \rho _{T,L}(\omega ,{\mathbf q}) \mathrm{d}\omega , \end{aligned}$$

with the spectral function being antisymmetric around the real-time frequencies origin $\rho (-\omega )=-\rho (\omega )$. From the perturbative study of gluon spectral functions in Landau gauge at $T=0$ it has been deduced (see [40, 46] for the explicit computations) that the following zero-area sum rule holds:

10 $$\begin{aligned} \int _{0}^\infty \omega \rho _{T,L}(\omega ,{\mathbf q})\mathrm{d}\omega =0. \end{aligned}$$

Since its derivation relies only on the asymptotics of the spectrum at large momenta, it applies also at finite temperature and clearly spells out that positivity violation of the spectrum is a manifest property. Equation (10) has been used and checked in continuum computations in many instances in the literature; see e.g. [19, 21, 23, 40]. On the other hand, since the derivation of Eq. (10) requires access to asymptotically large momenta, it can only hold approximately on the lattice. The sum rule emerges in the continuum limit, which can be demonstrated formally by inserting the lattice UV cutoff as upper limit in the superconvergence relation in e.g. Ref. [46].

Inverting Eq. (9) using the simulated lattice correlator data, in order to obtain the spectral functions, represents a well known ill-posed problem, which we will attack via the use of Bayesian inference as laid out in detail in the next section.

## Numerical methods

### Lattice simulations and gauge fixing

The present study works with lattices that feature four dynamical quark flavors, u, d, s and c. They represent a subset of configurations, which were generated by the tmfT collaboration originally for the study of QCD thermodynamics [53] in the presence of a heavy doublet of quarks (third and fourth quark species). The dynamical quarks are implemented using so-called twisted-mass actions, which take the following form for the light sector:

11 $$\begin{aligned} S^l_f[U,\chi _l,\overline{\chi }_l]= & {} \sum _{x,y} \overline{\chi }_l(x) [\delta _{x,y} -\kappa D_{\mathrm {W}}(x,y)[U] \nonumber \\&+\, 2 i \kappa a \mu _l \gamma _5 \delta _{x,y} \tau _3 ] \chi _l(y), \end{aligned}$$

and the heavy sector

12 $$\begin{aligned} S^h_f[U,\chi _h, \overline{\chi }_h]= & {} \sum _{x,y} \overline{\chi }_h(x) [ \delta _{x,y} - \kappa D_W(x,y)[U] \nonumber \\&+\, 2 i \kappa a \mu _{\sigma } \gamma _5 \delta _{x,y} \tau _1 + 2 \kappa a \mu _{\delta } \delta _{x,y} \tau _3 ] \chi _h(y),\nonumber \\ \end{aligned}$$

respectively, where the $\tau _i$ are the Pauli matrices in doublet (flavor) space. The term $D_{W}[U]$ denotes the standard gradient term for Wilson fermions

$$\begin{aligned} D_{W}[U] = \frac{1}{2 a} [ \gamma _\mu ( \nabla _\mu + \nabla ^{*}_\mu ) - \nabla ^{*}_\mu \nabla _\mu ] \end{aligned}$$

and $\kappa _l=(2 a m_{0,l} + 8 r)^{-1}$ represents the usual hopping term with $r=1$.

The gauge field degrees of freedom are governed—apart from the fermion backreaction—by an improved Iwasaki action

13 $$\begin{aligned} S_g[U]= & {} \beta \left( c_0 \sum _{P} \left[1 - \frac{1}{3} {\mathfrak {Re}}\, \mathrm{Tr}\left( U_{P} \right) \right]\right. \nonumber \\&\left. +\, c_1 \sum _{R} \left[1 - \frac{1}{3} {\mathfrak {Re}}\,\mathrm{Tr}\left( U_{R} \right) \right]\right) \end{aligned}$$

with ($c_0 = 3.648$ and $c_1 = -0.331$), where the sum (*P*) contains all plaquettes and the sum (*R*) all planar rectangles.

The light doublet $\chi _l = (\chi _u,\chi _d)$ in the twisted basis is related by a chiral rotation to the doublet $\psi ^{\mathrm{phys}} = (\psi _u,\psi _d)$ in the physical basis

$$\begin{aligned} \psi ^{\mathrm{phys}}_l = e^{i \omega _l \gamma _5 \tau _3/2} \chi _l \qquad \overline{\psi }^{\mathrm{phys}}_l = \overline{\chi }_l e^{i \omega _l \gamma _5 \tau _3/2} \end{aligned}$$

with the twisting angle $\omega _l$. Twisted-mass light fermions are taken at maximal twist, if the bare untwisted mass $m_{0,l}$ is tuned to its critical value $m_\mathrm{crit}$. When $|m_{0,l} - m_\mathrm{crit}| \rightarrow 0$, the twisting angle $\omega _l \rightarrow \frac{\pi }{2}$ (maximal twist). This fixes the twisted basis.

A similar rotation

$$\begin{aligned} \psi ^{\mathrm{phys}}_h = e^{i \omega _h \gamma _5 \tau _3/2} \chi _h \qquad \overline{\psi }^{\mathrm{phys}}_h = \overline{\chi }_h e^{i \omega _h \gamma _5 \tau _3/2} \end{aligned}$$

relates the two bases in the heavy sector. $\kappa _h=(2 a m_{0,h} + 8 r)^{-1}$ (with $r=1$) is the hopping parameter for heavy quarks. Again, $\omega _h \rightarrow \frac{\pi }{2}$ if $|m_{0,h} - m_\mathrm{crit}| \rightarrow 0$.

An economic procedure dealing with the $N_f=2+1+1$ case consists in the choice $a m_{0,l} = a m_{0,h} =\frac{1}{2 \kappa } -4$ with a common hopping parameter. Tuning to maximal twist means tuning $\kappa = \kappa _\mathrm{crit}(\beta )$. The critical $\kappa$ corresponds to the vanishing of the PCAC light-quark mass $m_\mathrm{PCAC}$ and is determined as a function of $\beta$ at zero temperature [54].

The bare light-quark ($\mu _l$) twisted-mass parameter (in the first doublet) and the two bare heavy-quark twisted-mass parameters $\mu _\sigma$ and $\mu _\delta$ (in the second doublet) also need to be tuned (as functions of $\beta$) at zero temperature to stay on a line of constant physics, defined by the “pion mass” and by matching masses of hadrons containing strange and charm quarks. For light hadrons this has been performed for the first time for $\beta =1.90$ and $\beta =1.95$ in Ref. [54].

The bare twisted-mass parameters $\mu _{\sigma }$ and $\mu _{\delta }$ are related to the renormalized strange and charm quark masses,

$$\begin{aligned} (m_s)_R= & {} Z_P^{-1} \left( \mu _{\sigma } - \frac{Z_P}{Z_S} \mu _{\delta } \right) (m_c)_R\\= & {} Z_P^{-1} \left( \mu _{\sigma } + \frac{Z_P}{Z_S} \mu _{\delta } \right) , \end{aligned}$$

with the renormalization constants $Z_P$ and $Z_S$ of the pseudoscalar and scalar quark densities.

For a more detailed description of the simulation setup see Refs. [54, 55].

The tmfT collaboration has adopted three parameter sets for their finite-temperature studies from the zero-temperature ensembles used by the ETMC collaboration (under the names A60.24, B55.32 and D45.32 defined in Ref. [55]). In Ref. [56] these ensembles have been calibrated with the help of the baryon spectrum, and we adopt these results for the lattice spacing. The set of $\beta$ values is fixed (according to the fixed-scale approach) and has been extended to include $\beta =1.90$ (A), $\beta =1.95$ (B) and $\beta =2.10$ (D). For example, the $T=0$ nomenclature “A60.24” indicates, besides the $\beta$ value, a lattice size $24^3\times 48$ for zero temperature and a light twisted-mass parameter $a \mu _l = 0.0060$. The corresponding physical lattice spacings and pion masses $m_{\pi ^{\pm }}$, together with the resulting deconfinement cross-over temperatures are listed in Table 1. The tmfT nomenclature refers to (apart from the $\beta$ value) to the approximate pion mass only. The temperature is varied by changing $N_\tau$.

**Table 1:** Properties of the three sets of finite-temperature ensembles used in our study, among them the deconfinement cross-over temperature $T_{\mathrm {deconf}}$ (defined by the Polyakov loop susceptibility)

ETMC ens. ($T=0$)	A60.24	B55.32	D45.32
tmfT ens. ($T\ne 0$)	A370	B370	D370
$\beta$	1.90	1.95	2.10
$a \, [\text {fm}]$	0.0936	0.0823	0.0646
$m_\pi \, [\text {MeV}]$	364(15)	372(17)	369(15)
$T_{\mathrm {deconf}}\, [\text {MeV}]$	202(3)(0)	201(6)(0)	193(13)(2)
$N_\tau =N_{q_4}\,\mathrm{range}$	4–14	10–14	4–20

To compute the gluon correlation functions (5) and (6), each generated configuration needs to be fixed to Landau gauge. This corresponds to the following discretized local condition:

14 $$\begin{aligned} \nabla _{\mu }A_{\mu }= \sum _{\mu =1}^{4} \left( A_{\mu }(x+\hat{\mu }/2) - A_{\mu }(x-\hat{\mu }/2) \right) = 0 \end{aligned}$$

on the gauge fields defined from the link variables as

15 $$\begin{aligned} A_{\mu }(x+\hat{\mu }/2)= \frac{1}{2iag_{0}}(U_{x\mu }-U_{x\mu }^{\dagger })\mid _\mathrm{traceless}. \end{aligned}$$

This condition may be fulfilled by iteratively applying local gauge transformations $g_{x}$

16 $$\begin{aligned} U_{x\mu } {\mathop {\mapsto }\limits ^{g}} U_{x\mu }^{g} = g_x^{\dagger } U_{x\mu } g_{x+\mu }, \quad g_x \in SU(3), \end{aligned}$$

in order to maximize the functional

17 $$\begin{aligned} F_{U}[g]= \dfrac{1}{3} \sum _{x,\mu } {\mathfrak {Re}}\,\mathrm{Tr}\left( g_{x}^{\dagger } U_{x\mu } g_{x+\mu } \right) . \end{aligned}$$

We consider a configuration to have reached a (local) extremum if the global deviation is less than

18 $$\begin{aligned} \max _{x}{\mathfrak {Re}}\,\mathrm{Tr}[\nabla _{\mu }A_{x\mu }\nabla _{\nu }A_{x\nu }^{\dagger }]< 10^{-13} . \end{aligned}$$

This procedure has been carried out by means of the cuLGT library [57], which we have adapted for the use with lattice configurations in the ILDG format.

Subsequently we transform the gauge fields (15) into Fourier space, where the lattice momenta are defined as

19 $$\begin{aligned} k_\mu a=\frac{\pi n_{\mu }}{N_{\mu }}, \quad n_\mu \in (-N_{\mu }/2, N_{\mu }/2]. \end{aligned}$$

They are related to physical momenta via

20 $$\begin{aligned} q_{\mu }(n_{\mu }) = \frac{2}{a} \sin \left( \frac{\pi n_{\mu }}{N_{\mu }}\right) . \end{aligned}$$

### Bayesian spectral reconstruction

The extraction of gluon spectral functions from simulated correlation functions poses an inherently ill-defined problem. Our task is to select a unique continuous function to reproduce a finite and noisy set of datapoints. In order to be able to resolve the anticipated peaked features in the spectrum one discretizes it along real-time frequencies $\omega$ with *O*(1000) bins, while as shown in Table 1 the number of available correlator points ranges over $N_{q_4}\in [4\ldots 20]$. Hence inverting a discretized Eq. (9)

21 $$\begin{aligned} D_i^\rho =\sum _{l=1}^{N_\omega } \varDelta \omega _l K_{il} \rho _l,\qquad i\in [0,N_{q_4}], \quad {N_\omega \gg N_{q_4}} \end{aligned}$$

via a naive $\chi ^2$ fit of the $\rho _l$ parameters would yield an infinite number of degenerate solutions.

In the present case of gluon spectra the severity of the ill-posedness of the inverse problem is worsened by the non-positivity of the gluon spectrum. Note that even if the sum rule (10) is implemented in the reconstruction this additional difficulty is not cured. A formal analysis of this issue is currently work in progress [59].

Just as in the positive-definite case, Bayes theorem can provide us with a viable strategy to regularize the otherwise ill-defined problem. It states that the probability of a test spectral function $\rho$ to be the correct spectrum, given measured data and further, so-called prior information (*I*) is proportional to the product of two terms

22 $$\begin{aligned} P[\rho |D,I]\propto P[D|\rho ,I]P[\rho |I]. \end{aligned}$$

This expression follows from the multiplication theorem of conditional probabilities and formally allows prior information *I* to influence both terms on the right hand side. The first $P[D|\rho ,I]=\mathrm{exp}[-L]$ refers to the likelihood probability, which in our case is related to the $\chi ^2$ fitting functional. The likelihood *L* measures the quadratic distance between the correlator points corresponding to the test function $\rho$ and the simulated data $D_i$

23 $$\begin{aligned} L=\frac{1}{2}\sum _{i,j=1}^{N_{q_4}}(D_i-D^\rho _i)C_{ij}^{-1}(D_j-D^\rho _j), \end{aligned}$$

where $C_{ij}$ refers to the usual covariance matrix of the simulated $D_i$’s. Prior information enters implicitly, as we will only accept spectra for which $L=N_{q_4}$, which corresponds to our prior knowledge that the correct spectrum, sampled randomly with Gaussian noise, would on average lead to such a value of the likelihood. Note that *L* by itself possesses $N_\omega -N_{q_4}$ flat directions, which in the Bayesian approach are regularized by introducing the prior probability $P[\rho |I]=\mathrm{exp}[\alpha S (\omega )]$.

This second term encodes further information we possess about the spectrum, beyond the simulation data, which may take the form of a smoothness condition, a sum rule or in the case of hadronic spectra refer to positive-definiteness. Prior information enters in two ways: on the one hand the functional form of *S* itself encodes part of that information, on the other hand *S*[*m*] conventionally depends on a function $m(\omega )$ called the default model. By definition *m* corresponds to the correct spectrum in the absence of data, i.e. it represents the unique extremum of *S*. Since Eq. (10) may not be exact at finite lattice spacing we refrain from strictly enforcing the sum rule in the following. As we, however, expect that the area under the spectrum will be close to zero and wish to use an otherwise unbiased default model we choose the function $m(\omega )$ to vanish identically.

Since for gluon spectra we may not assume positive-definiteness, standard regulators, such as those of the MEM $S_\mathrm{SJ}$ [41] or the standard BR method [43]

24 $$\begin{aligned} S_\mathrm{BR}=\int \mathrm{d}\omega \left( 1- \frac{\rho (\omega )}{m(\omega )} + \mathrm{log}\left[ \frac{\rho (\omega )}{m(\omega )} \right] \right) \end{aligned}$$

are not applicable. While the Shannon Jaynes entropy of the MEM has been generalized to treat non-positive-definite spectra [42], which approach requires us to a priori choose a decomposition of the spectrum in positive and negative components. Since the information of where the spectrum starts to become negative is among those we wish to learn from such a reconstruction, we refrain from following this strategy. On the other hand other regulators, such as quadratic ones have been proposed [20]. Unfortunately we have found in previous studies that these very strongly pull the final result towards the default model, especially if only a relatively small number of correlator points are available. This may not allow the information encoded in the simulation data to manifest itself in a satisfactory manner.

Instead we will deploy here a recently developed regulator $S_\mathrm{BR}^g$ [14], which shares many of the advantageous analytic properties of that used in the standard BR method

25 $$\begin{aligned} S_\mathrm{BR}^g= & {} \int \mathrm{d}\omega \left( -\frac{|\rho (\omega )-m(\omega )|}{h(\omega )}\right. \end{aligned}$$

26 $$\begin{aligned}&\left. +\,\mathrm{log}\left[ \frac{|\rho (\omega )-m(\omega )|}{h(\omega )}+1 \right] \right) . \end{aligned}$$

Since now $\rho$ and *m* can both take on the value zero, one uses a different measure for deviation between the default model and the spectrum $r_l=|\rho _l-m_l|/h_l$. The function $h_l$, absent in the standard BR regulator, corresponds to an additional default-model-like function, which encodes the confidence we have in $m_l$. $S_\mathrm{BR}^g$ does not require us to choose a decomposition a priori, since the role of $m(\omega )$ is unchanged. Furthermore it imprints the form of the default model relatively weakly onto the end results, as its curvature in the region where $\rho$ differs significantly from *m* is smaller than in the $S_{SJ}$ or the quadratic prior (as discussed in [14]).

Both the choice of *m* and of *h* contribute to the systematic uncertainties of the reconstructed spectrum. Thus their values need to be varied to ascertain, which parts of the spectrum are fixed predominantly by the correlator data. Note also that a hyper-parameter $\alpha$ has been introduced in the definition of the prior probability, taking into account that we may weight the influence of data and prior information independently from each other. The analytic form of $S_\mathrm{BR}^g$ allows us to integrate $\alpha$ out in a straight-forward fashion, assuming full ignorance about its values $P[\alpha ]=1$

27 $$\begin{aligned} P[\rho |D,I,m]\propto P[D|\rho ,I]\int _0^{\infty } \mathrm{d}\alpha P[\rho | m,\alpha ] P[\alpha ]. \end{aligned}$$

Once $m(\omega )$ and $h(\omega )$ are specified we have to carry out a numerical search for the most probable Bayesian spectrum according to

28 $$\begin{aligned} \left. \frac{\delta P[\rho |D,I]}{\delta \rho } \right| _{\rho =\rho ^\mathrm{Bayes}} = 0, \end{aligned}$$

which, as laid out above, consists of a competition between reproducing the simulation data and conforming to prior information. Even though we do not restrict the space of basis functions for this search, i.e. the optimization is performed in $N_\omega \gg N_{q_4}$ degrees of freedom, we have confirmed that different starting points lead to the same extremum. The underlying reason is that $S_\mathrm{BR}^g$ fulfills the conditions required in the proof of uniqueness of the Bayesian solution given in [41].

For the reconstructions to be presented in Sect. 4 we deploy the generalized BR method on a frequency grid $\omega \in [10^{-3},100]$ GeV divided in $N_\omega =2000$ bins. To ensure that the convolution in (9) evaluated over such a relatively large frequency interval does not suffer from numerical precision losses, the computations are carried out using 512 bit arithmetic. In order to further improve the stability of the numerical optimization task, we deploy the following prescription for the kernel: $K(q_4,\omega )=2\mathrm{ArcTan}(\omega )\omega /(q_4^2+\omega ^2)$. This means that instead of $\rho$ itself, we reconstruct the function $\rho (\omega )/\mathrm{ArcTan}(\omega )$. To plot and investigate the spectra we then divide out the arctan term. This rewriting of Eq. (9) enforces that the spectrum $\rho (\omega )$ vanishes at $\omega =0$. In addition by using the lattice momenta $q_4$ in the kernel we take into account that the UV region of the Matsubara frequencies on the lattice is affected by the finite lattice spacing, making the reconstruction algorithm converge more quickly.

Since the zero-area sum rule Eq. (10) is derived from the RG running at asymptotically large frequencies not present on a lattice, it is only estabilished in the continuum limit. Thus we here refrain from using it to further constrain the reconstruction. As the sum rule emerges in the continuum limit, we, however, set our initial default model $m(\omega )$ to zero, while the confidence function $h(\omega )$ is set to unity.

The robustness and reliability of the spectral reconstruction is ascertained in the following way. There exist two intertwined sources of uncertainty in our approach: on the one hand statistical uncertainty arises from the finite precision inherent in our lattice simulation correlators. On the other hand, due to the fact that only a finite number of datapoints are available, we incur a systematic uncertainty from the necessity to choose the default-model functions $m(\omega )$ and $h(\omega )$. How strongly the end result suffers from either statistical or systematic uncertainty can be explicitly checked by performing a Jackknife reweighting, where the spectral reconstruction is performed multiple times on a subset of the available simulation data for the former or by varying the values of the default models for the latter.

In Sect. 4 we will deploy a ten-bin Jackknife. It shows that the available signal to noise ratio in the lattice correlators is sufficiently high for statistical uncertainty to play only a minor role. Instead, as is probed by varying the value of the constant default model between $m=\{-2,0,2\}$ and varying the confidence function $h=\{1,2\}$, we find that systematic uncertainty dominates the error budget which will be presented in the sections below as transparent error-bands on the reconstructed spectra. Changing the default model by an absolute value of two might at first appear as a rather small change, but due to the relatively large range of frequencies up to $\omega _\mathrm{max}=100$ GeV it represent a significant change in the area covered by the function. It is the area under the default model to which the final reconstruction result is susceptible, so that if reconstructed features remain unchanged even after changing the default-model area significantly, we can consider them as robustly encoded in the underlying correlator data.

We note that Bayesian approaches, such as our BR method or the Maximum Entropy Method, provide for an additional measure of robustness. This measure relies on the assumption of a highly peaked posterior and the further assumption that the posterior can be approximated by a Gaussian (see e.g. [41]). Then the integrated curvature of the minimization functional

29 $$\begin{aligned} Q[\rho ,m,h]=\mathrm{Log}[ P[D|\rho ,I]\int _0^{\infty } \mathrm{d}\alpha P[\rho | m,\alpha ] ] \end{aligned}$$

may serve as a measure of reliability

30 $$\begin{aligned} \langle \delta \rho ^2 \rangle _I \approx - \int _I \mathrm{d}\omega \mathrm{d}\omega ^\prime (\delta ^2Q/\delta \rho ^2)^{-1}\Big /\int _I \mathrm{d}\omega \mathrm{d}\omega ^\prime . \end{aligned}$$

In essence it describes how shallow the global extremum is, which defines the most probable Bayesian spectrum. In practice we have found that it often underestimates the uncertainty of the reconstruction when compared to the explicit variation of the default model and the Jackknife analysis. Since we are able to perform both Jackknifing and a variation of default models we therefore refrain from using the curvature criterion.

## Correlation functions

This section is devoted to a presentation and discussion of the computed gluon correlation functions in Landau gauge on $N_f=2+1+1$ tmfT lattices. All figures below that show correlator data include statistical error estimates. Since the statistics of the tmfT ensembles is relatively high and the gluon propagator in imaginary frequencies does not show an exponential falloff, its signal to noise ratio is good enough so that our errorbars are mostly smaller than the point size used for plotting.

At first sight the availability of several different lattice spacings based on the three $T=0$ ETMC ensembles invites the attempt of a continuum extrapolation. The main purpose of the $T>0$ ensembles was the study of thermal bulk quantities, such as the equation of state and a fixed-scale approach that had been adapted, the present ensembles, however, do not resolve the same temperatures with different lattice spacings. This in turn requires an additional intermediate interpolation in temperature and momenta for extrapolating the correlators. We have tested its viability and found it to be insufficient, since the underlying temperature grid is too coarse. Thus in the following we will concentrate mainly on the *D*370 ensemble (Table 2), which is both closest to the continuum and spans the broadest temperature range. Selected results from the other ensembles at different $\beta$ values will be considered where it provides additional qualitative insight (Table 1).

**Table 2:** Grid sizes and temperatures in the D370 ensembles used for the computation of the correlation functions below. $N_{\mathrm{meas}}$ refers to the number of available correlator measurements

*D*370 $N_{\tau }$	4	6	8	10	11	12	14	16	18	20
*T* MeV	762	508	381	305	277	254	218	191	170	152
$N_s$	32	32	32	32	32	32	32	32	40	48
$N_{\mathrm {meas}}$	310	400	120	410	420	380	790	610	590	280

We will connect to the current literature by carrying out Gribov–Stingl fits to the momentum space correlators and comparing with published results in both quenched QCD, as well as with more recent $N_f=2$ dynamical QCD simulations. For future reference we also provide a visualization of the zero Matsubara frequency correlators $D(0,|\mathbf {q}|^2)$, which in the literature have been used to benchmark functional computations in both Yang–Mills and $N_f=2$ QCD.

In preparation of the reconstruction of spectral functions via Bayesian inference we will consider the correlators at a fixed spatial momentum $D(q_4,|\mathbf {q}|)$ along the different available imaginary frequencies $q_4$ on the lattice. In the continuum, the finite extent of the Euclidean time axis leads to equidistantly spaced Matsubara frequencies $\mu _n=2\pi T n$, at which the imaginary frequency correlator is routinely evaluated. In the presence of a finite lattice spacing the discrete Fourier transform connecting Euclidean times and imaginary frequencies leads to a artifacts close to the edge of the Brillouin zone, so that the physical momenta $q_4$ are not any longer spaced at the same distance. As we carry out the reconstruction using the kernel based on $q_4$ we also plot the correlators in these physical imaginary frequencies. Since temperature scans are carried out using a fixed-scale prescription, the maximum available imaginary frequency remains constant and it is the spacing between values of $q_4$ that grows with increasing *T*.

**Table 3:** Best fit parameters and their uncertainty for the Gribov–Stingl fits, applied to the longitudinal (top five rows) and the transversal (bottom five rows) correlator at $\beta =2.10$

$\text {Stingl fits}$ $T/T_C$	3.95	2.63	1.98	1.58	1.44	1.32	1.13	0.99	0.88	0.79
$D_L$ – $d/a^2$	0.25(2)	0.30(2)	0.48(3)	0.68(4)	0.75(5)	0.82(6)	0.92(6)	0.96(7)	1.25(10)	1.36(9)
$D_L$ – $r^2 a^2$	0.87(19)	0.60(9)	0.34(4)	0.23(3)	0.20(2)	0.17(2)	0.14(2)	0.12(2)	0.096(10)	0.086(7)
$D_L$ – $c / a^2$	1132 (82)	946(47)	642(28)	478(26)	436(24)	408(25)	366(22)	349(22)	281(18)	262(15)
$D_L$ – $b a^2$	0.88(2)	0	0	0	0	0	0	0	0	0
$D_L$ – $\chi ^2/\mathrm{d.o.f.}$	0.78	0.74	0.75	0.83	0.85	0.84	0.86	0.86	0.90	0.91
$D_T$ – $d/a^2$	0.28(1)	0.35(2)	0.52(3)	0.68(5)	0.75(6)	0.80(8)	0.87(8)	0.91(9)	0.99(4)	0.85(7)
$D_T$ – $r^2 a^2$	0.21(2)	0.26(2)	0.19(2)	0.15(2)	0.13(2)	0.12(2)	0.11(2)	0.11(2)	0.11(3)	0.12(1)
$D_T$ – $c / a^2$	806(20)	698(23)	523(27)	427(26)	394(29)	374(27)	348(27)	336(30)	324(9)	357(23)
$D_T$ – $b a^2$	0.411(4)	0.12(2)	0	0	0	0	0	0	0	0
$D_T$ – $\chi ^2/\mathrm{d.o.f.}$	0.80	0.84	0.84	0.87	0.89	0.88	0.88	0.90	0.89	0.88

### Zero-Matsubara frequency correlators

Let us start with an inspection of the raw correlator data obtained from the lattice, evaluated at vanishing Matsubara frequencies. It has been found that both longitudinal and transversal correlators may be described at low and intermediate momenta with a simple formula [15, 27, 29], attributed to Gribov [16] and Stingl [17]. It reads

31 $$\begin{aligned} D^\mathrm{GS}_{L/T}(q)=\frac{c(1+dq^{2n})}{(q^2+r^2)^2+b^2} \end{aligned}$$

where in the following the parameter *n* is set to unity, corresponding to the ’quasi-particle’ scenario, as detailed below. The asymptotic behavior of the correlators for large momenta is known perturbatively and exhibits powers of logarithms [40, 46], which we here neglect. In turn this simple description is expected to fail eventually as we move to higher momenta.

**Fig. 1:**
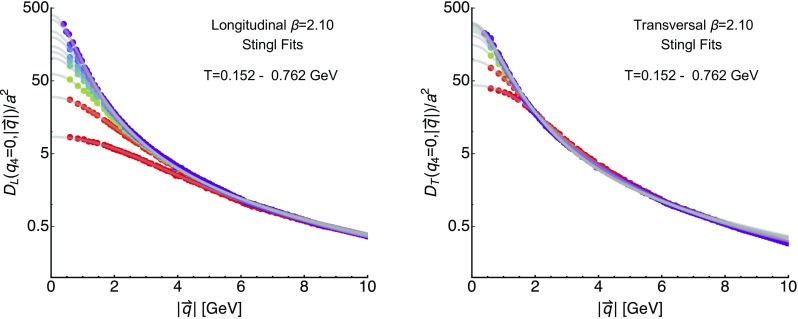
The longitudinal (left) and transversal (right) gluon correlators at $\beta =2.10$ evaluated at vanishing imaginary frequency $q_4=0$ but finite spatial momenta $|\mathbf {q}|$ for different temperatures $T=152\ldots 762$ MeV. The gray solid lines correspond to the results of the Gribov–Stingl fits

**Fig. 2:**
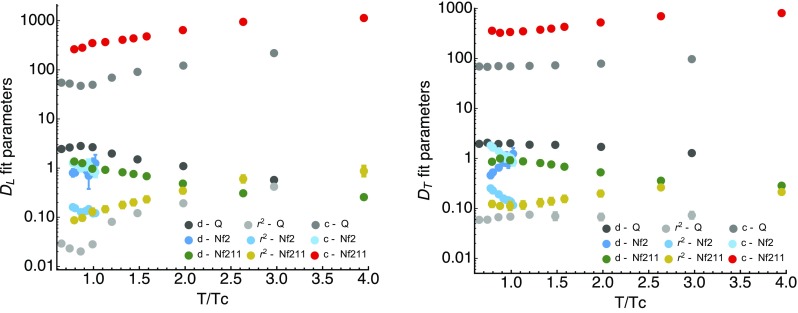
Comparison of the Stingl-fit parameters $c / a^2$(top points), $d/a^2$ (middle points) and $r^2 a^2$ (bottom points) obtained from quenched QCD (gray shades), from $N_f=2$ full QCD (light blue shades) and our $N_f=2+1+1$ full QCD (red, yellow, green). Note that the results from quenched QCD and $N_f=2+1+1$ show qualitatively consistent behavior, while the short temperature range spanned by the $N_f=2$ study at first sight shows opposite behavior. This difference is related to the fact that the former two are carried out in a fixed-scale approach, while the latter are carried out in a fixed box approach

The individual parameters inform us about vital aspects of the physics encoded in the correlator. Most easily this is seen for $b=0$ and $d=0$, where the form is equivalent to $D^\mathrm{GS}_{L/T}(q)=\frac{\kappa _1}{(q^2+r^2)^2}$, which encodes a pair of complex-conjugate poles that may be associated with a stable quasi-particle. The dynamically generated mass of such an excitation is then related to the value of the parameter $m=r$. The presence of the parameter $b\ne 0$, the so-called Gribov–Zwanziger term, introduces a modification to the pole structure, i.e. destabilizing the quasi-particle, while the $d\ne 0$ term introduces the possibility for a genuine zero of the analytically continued propagator when the two terms in the numerator cancel. Since here we deploy a fixed-scale approach, the renormalization of the correlators remains unchanged between different temperatures. Otherwise, since the correlator renormalizes multiplicatively, the factor *c* would experience direct contributions from changes in scale.

To determine the parameter values of Eq. (31) we fit the unrenormalized longitudinal and transversal correlators $D_{L/T}(0,|\mathbf {q}|)$ on the D370 ensembles in the range above $|q|\sim 0.6$ GeV up to 6 GeV. Except for the highest in the longitudinal and the two highest temperatures in the transversal sector, the fits show a clear preference for a vanishing $b=0$. The resulting best fit values are listed in Table 3, the data and corresponding fit functions are plotted in Fig. 1. With consistent $\chi ^2/d.o.f.$ values smaller than unity we find that the Gribov–Stingl form works well in the momentum regime considered here. The fit continues to describe the longitudinal data well even up to $|q|\sim 10$ GeV, while it seems to deviate from the transversal correlator sizably above $|q|\sim 8$ GeV. One possible reason may be an earlier emergence of the logarithmic corrections to the rational form in the transversal sector.

Already by eye significant differences in the temperature dependence of the infrared behavior of the longitudinal and transversal correlator are visible in Fig. 1. The former shows a much stronger change to smaller values with increasing *T* compared to the latter, manifest also in the fitted $r^2$ and *c* values. Since the $q_4=0$ correlator at vanishing spatial momentum (in that order of limits) provides insight on the inverse screening mass of the theory, screening in the longitudinal (electric) sector, as expected, is more efficient than in the transversal (magnetic) sector. We will come back to the determination of the quasi-particle masses in the context of Bayesian spectral reconstructions in the following sections.

Let us compare the fit values found here with those obtained in previous studies using either Szymanzik improved quenched QCD [29], or full QCD [27], with $N_f=2$ twisted-mass flavors of light fermions (B12 dataset with $m_\pi =398$ MeV) in Fig. 2. We note that the lattice study of Yang–Mills theory was also carried out in a fixed-scale approach, while for $N_f=2$ the temperature was changed by varying the lattice spacing. Since all Gribov–Stingl fits were performed on the unrenormalized correlators, we may expect that similar trends only emerge within the same approach. And indeed, what we find is that the results from quenched QCD and our $N_f=2+1+1$ lattices show a consistent behavior. In the high-temperature phase $d/a^2$ decreases, while $r^2 a^2$ and $c/a^2$ increase monotonously. While not plotted as additional dataset, the behavior of the mass parameter *r* is also consistent with the results obtained from quenched QCD in [18].

**Fig. 3:**
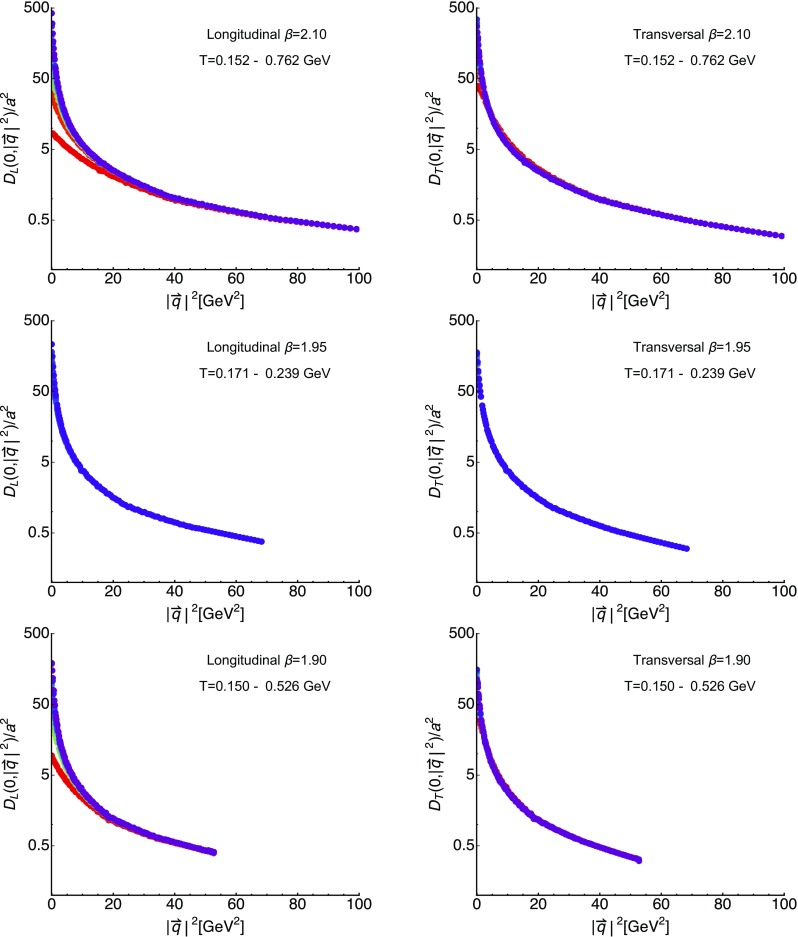
For completeness we show here the longitudinal (left) and transversal (right) gluon correlators at $\beta =2.10$ (top),$\beta =1.95$ (middle) and $\beta =1.90$ (bottom) evaluated at vanishing imaginary frequency $q_4=0$ but finite spatial momenta $|\mathbf {q}|^2$ for different temperatures

**Fig. 4:**
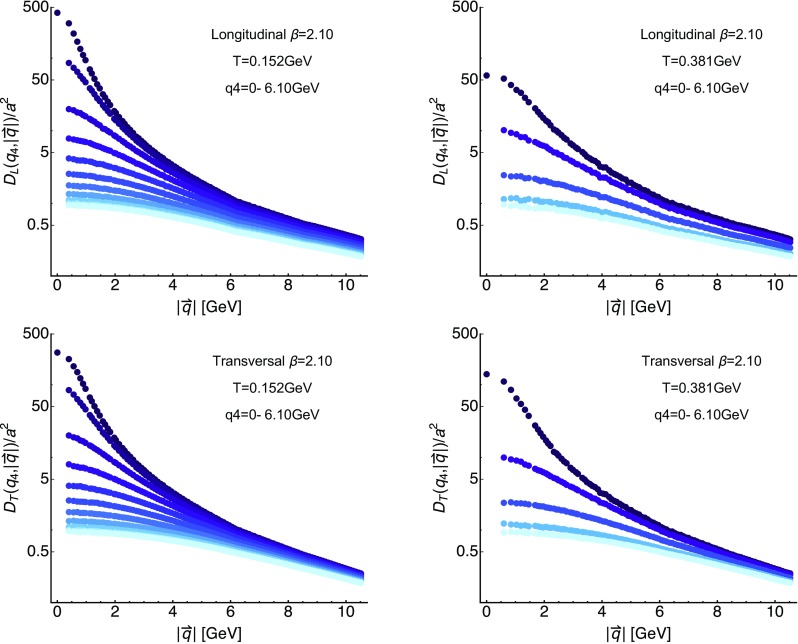
The longitudinal (top row) and transversal (bottom row) gluon propagators at $\beta =2.10$ evaluated at both finite imaginary frequency $q_4$ and spatial momentum $|\mathbf {q}|$. We show the $|\mathbf {q}|$ dependence at fixed $q_4$ for the lowest $T=0.152$ GeV (left) and a high $T=0.381$ GeV (right). The color coding assigns darkest colors to the lowest value of $q_4$

The genuine phase transition present in *SU*(3) manifests itself in a change of behavior in all longitudinal fit parameters around $T=T_C$, while for $N_f=2+1+1$ the cross-over does not appear to induce a similar feature. On the other hand our $N_f=2+1+1$ data stops shortly below the deconfinement cross-over transition temperature and we may just not be able to observe a similar change in the parameter behavior without extending the ensembles to smaller temperatures. The transversal sector shows less variation in the parameters, as expected from a naive inspection by eye of the correlators themselves. Neither the quenched data, nor our $N_f=2+1+1$ data shows significant changes around $T=T_C$.

For completeness we have also included the fit results from a study based on $N_f=2$ full QCD in Fig. 2 with light blue shades. That study spanned only a small region of temperatures around the phase transition and is based on a fixed box approach. Their fits to the unrenormalized correlators yield a very different behavior, essentially showing opposite trends to those observed in both quenched and our $N_f=2+1+1$ study. The multiplicative renormalization of the correlator itself will affect the constant *c*, as a renormalization of the mass can affect *r*. A reanalysis of the temperature dependence using $N_f=2$ data and appropriately renormalized correlators may thus be illuminating.

**Fig. 5:**
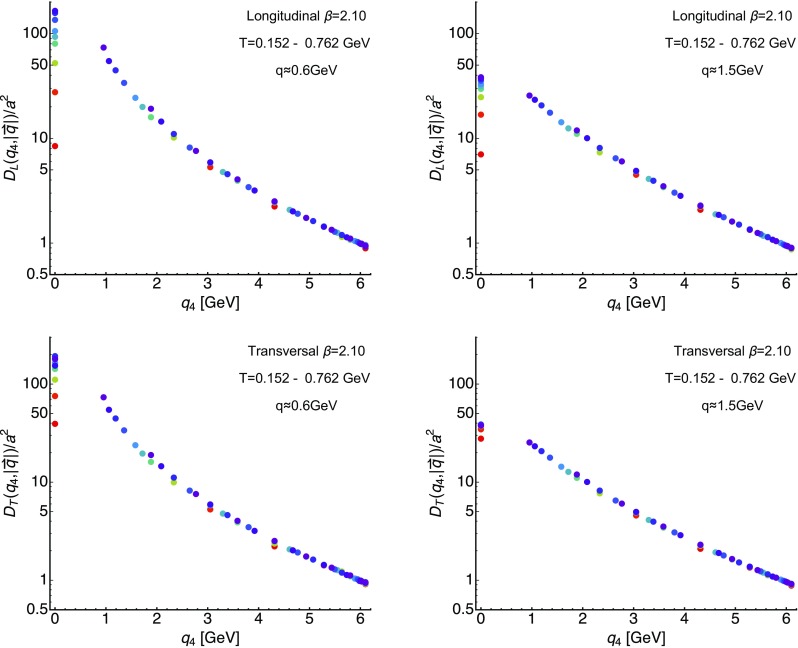
The longitudinal (top row) and transversal (bottom row) gluon propagators at $\beta =2.10$ evaluated along imaginary frequencies for the ten available temperatures among the ensembles of *D*370, i.e. $T=152\ldots 762$ MeV. The left panels contains correlators at $|\mathbf {q}|\approx 0.6$ GeV while the right panel shows those at $|\mathbf {q}|\approx 1.5$ GeV. Note that above the first finite Matsubara frequency all data points essentially line up on the same $T=0$ curve

As last item in this subsection we plot for completeness in Fig. 3 the full spatial momentum dependence of the longitudinal and transversal correlators at the different available values of $\beta =2.10,1.95,1.90$ (top, middle, bottom, respectively). They illustrate the approach of the correlators to their $T=0$ behavior at large momenta such that for $|\mathbf {q}|^2\gg T^2$ they take on the same values and at the highest momenta shown are virtually indistinguishable.

### Finite-Matsubara frequency correlators

We continue with an inspection of the finite Matsubara frequency correlators, several representative ones we have plotted in the panels of Fig. 4. The top row contains the longitudinal correlators, while the bottom row those of the transversal sector. In each panel there are $N_\tau /2$ curves corresponding to the resolved imaginary frequencies on the corresponding lattices vs. spatial momentum. A clear ordering in imaginary frequencies is present at all temperatures, with the values at higher $q_4$ being smaller than those at lower imaginary frequencies. Only at $q_4=0$ is the value at $|q|\rightarrow 0$ well defined, which is why only the top most data include this point.

More important for us, however, is the temperature dependence of the correlators along imaginary frequencies, as exhibited at fixed spatial momentum, since it is from these datapoints that we will eventually reconstruct the gluon spectral functions. In Fig. 5 these correlators are shown, with the top row containing the longitudinal ones and the bottom row containing the transversal ones. The left column houses the data for relatively small spatial momenta $q\approx 0.6~\hbox {GeV}$, while the right column contains those for $q\approx 1.5$ GeV. The temperature range spans from $T=0.152$ GeV (dark purple) to $T=0.762$ GeV (red).

**Fig. 6:**
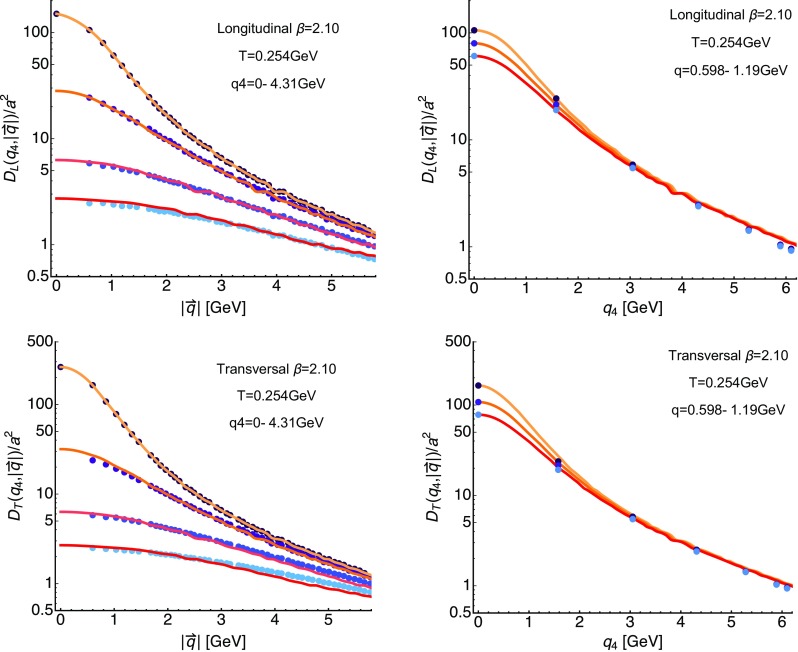
The longitudinal gluon propagators for $\beta =2.10$ evaluated at $T=254$ MeV. (left) Shown are correlators vs. spatial momenta at the first four available imaginary frequencies $q_4=0-4.31$ GeV. The orange curve represents a naive spline interpolation $\tilde{D}_{L/T}(|\mathbf {q}|)$ of $D_{L/T}(q_4=0,|\mathbf {q}|)$. The dark orange and red curves correspond to the interpolation evaluated using the assumption of *O*(4) invariance: $\tilde{D}_{L/T}(\sqrt{q_4^2 + |\mathbf {q}|^2})$. (Right) Use of the interpolation $\tilde{D}_{L/T}(|\mathbf {q}|)$ in order to reproduce the finite imaginary frequency behavior of the propagator (blue points). The solid curves show the *O*(4) evaluation $\tilde{D}_{L/T}(\sqrt{q_4^2 + |\mathbf {q}|^2})$. We can see that, while for small imaginary frequencies the *O*(4) ansatz works quite well, it starts to degrade as one approaches the boundary of the Brillouin zone due to breaking of rotational symmetry on the lattice

The first observation to be made is that indeed at vanishing Matsubara frequency both longitudinal and transversal correlators show clear distinguishable values at different temperatures. For the former the $q_4=0$ values are strictly ordered by temperature, decreasing in value as *T* increases, while for the latter the values first seem to rise below $T_C$ and then decrease above $T_C$. As expected from our previous inspection of the $q_4=0$ correlators, temperature effects in the transverse sector are milder than in the longitudinal sector.

While not surprising, it is important to note that, for finite imaginary frequencies, in particular above the first Matsubara frequency $\omega _1=2\pi T$, the datapoints all but collapse onto a single curve, which represents essentially $T=0$ physics. This phenomenon is even more pronounced at higher momenta, where temperature effects are naturally suppressed by the momentum scale. In anticipation of the extraction of gluon spectral functions this is a stark reminder of the difficulties involved. In essence we will attempt to extract from two or maximally three datapoints, which are temperature sensitive, the full in-medium modification of the gluon spectrum, a challenging proposition. To succeed, it then becomes necessary to determine the minute changes in the datapoints at higher $q_4$ with very good precision. On the other hand a significant improvement of the spectral reconstruction would result if the regime between the zeroth and first Matsubara frequency, where most of the temperature effects are hidden, could be resolved (first attempts in this direction have been reported in [58]).

At $T=0$ the conceptual restriction of a finite spacing of imaginary frequencies is non-existent and they may be as finely resolved as computationally feasible. In addition at $T=0$ the Euclidean correlation functions naturally exhibit an *O*(4) symmetry, which may be used to obtain the correlator values at finite imaginary frequencies by evaluating the correlator at zero imaginary frequencies, while appropriately shifting the finite spatial momentum $D(q_4,|\mathbf {q}|)\approx D(0,\sqrt{q_4^2+|\mathbf {q}|^2})$. At finite temperature compactified imaginary time starts to play a special role and *O*(4) invariance is not exact anymore. It may nevertheless provide a useful approximation and using spline interpolations along spatial momenta it has been verified in continuum computations. The *O*(4) scaling assumption there applies with less than 10% error up to the first Matsubara frequency and with even less error at the higher frequencies. In turn one may also attempt to access the regime between the zeroth and first Matsubara frequency, which promises improvements in the spectral reconstruction. This experience in the continuum has subsequently motivated the use of the *O*(4) ansatz in lattice studies; see e.g. [20].

### The *O*(4) scaling ansatz on the lattice

On the lattice the *O*(4) symmetry is already broken by the finite lattice spacing and the finite box. Therefore we set out here to investigate the validity of the *O*(4) scaling assumption for the longitudinal (top row) and transversal (bottom row) correlator in Fig. 6. In the left panels we set up a spline interpolation $\tilde{D}_{L/T}$ (solid yellow line) of the correlator $D_{L/T}$ for $\beta =2.10$ at $T=254$ MeV at vanishing imaginary frequencies (topmost points) along spatial momenta $|\mathbf {q}|$. The orange and red curves then correspond to this interpolation, evaluated according to $\tilde{D}_{L/T} (0,\sqrt{q_4^2+|\mathbf {q}|^2})$ at the first three finite available imaginary frequencies. We find that at $q_4\approx 2 \pi T$ the *O*(4) ansatz works acceptably well, starting from $|\mathbf {q}|\approx 1.5$ GeV$^2$. As expected from experience with continuum computations the *O*(4) scaling does not work as well for the transversal part at higher imaginary frequencies and starts to deviate from the simulated data already at around $|\mathbf {q}|=3$ GeV.

**Fig. 7:**
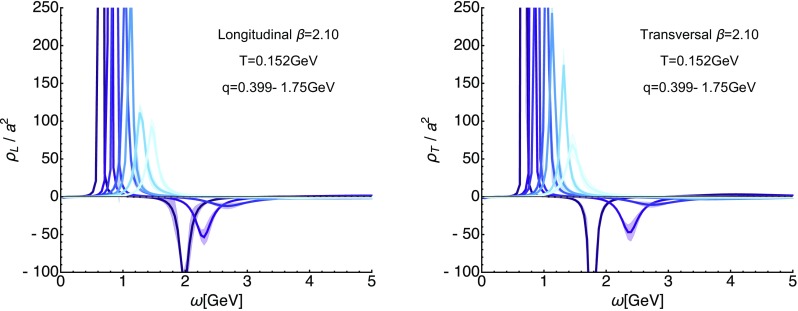
Reconstructed longitudinal (left) and transversal (right) gluon spectra on the $\beta =2.10$ (top) ensemble at the lowest available temperature $T=0.152$ GeV. The different curves denote a subset of spatial momenta at which correlator data is available. The *y*-axis is cut off to showcase the existence of negative contributions. One can clearly see a well defined lowest lying positive peak with a subsequent trough, which dies out towards higher frequencies. Error-bands arise from varying the default-model functions *m* and *h*

Different from the continuum, the finite lattice spacing manifests itself in the breaking of rotational symmetry, which affects the edge of the Brillouin zone most severely. It is then exactly at high spatial momenta where we see deviations from the *O*(4) ansatz appearing. In the right panels we use $\tilde{D}_{L/T}$ to evaluate the correlator along imaginary frequencies. We find that it provides a smooth curve, which for the four lowest finite imaginary frequencies, available on the lattice, lies quite close to the actual datapoints, while it starts to deviate again towards the edge of the Brillouin zone.

In conclusion, since for the spectral reconstruction very precise correlator data is required and we find that systematic uncertainties due to finite lattice spacing artifacts are manifest in the application of the *O*(4) ansatz to finite-temperature lattice gluon correlators, we do not deploy it further in this study. Instead we will only use the actual computed correlator values along discrete imaginary frequencies for the spectral reconstructions in the next section.

## Reconstructed spectral functions

We continue in this section by presenting the spectral functions reconstructed from the correlation functions discussed in the previous section via the generalized Bayesian BR approach. One central aspect in the determination of spectral functions from Euclidean data is to assess the unavoidable uncertainty in the end result, due to the inherently ill-posed nature of the reconstruction process. Two kinds of systematic errors are always present: those related to the finite number of available datapoints and those related to the finite precision of the lattice correlators. The latter is assessed via a Jackknife resampling procedure, the former via changing the underlying default model related input functions $m(\omega )$ and $h(\omega )$, as discussed in Sect. 2.2. That is, all figures depicting spectral functions in the following contain error-bands, which denote the variation due to those two factors.

**Fig. 8:**
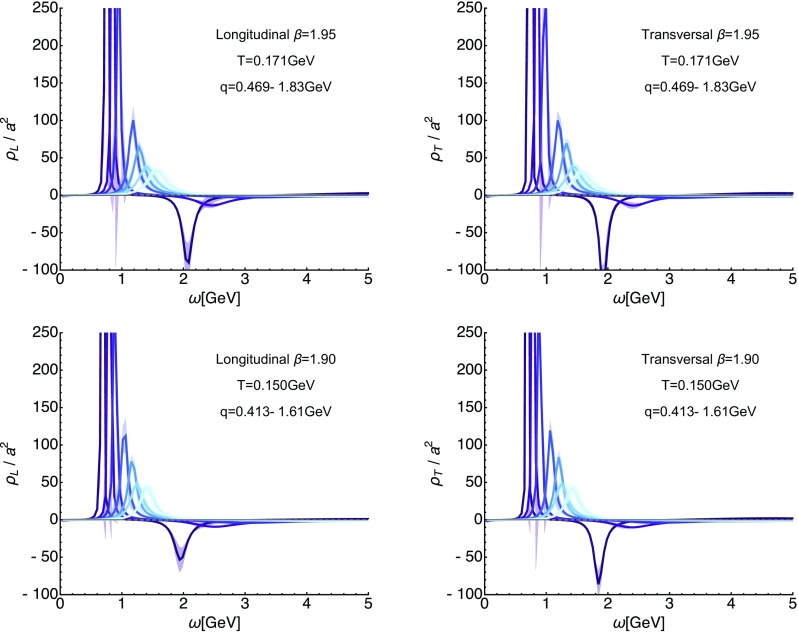
For completeness we show here the reconstructed longitudinal (left) and transversal (right) gluon spectra on the $\beta =1.95$ (top) and $\beta =1.90$ (bottom) ensembles at the lowest available temperatures. The different curves denote a subset of spatial momenta at which correlator data is available, lighter colors denote larger momenta

### Low-temperature spectral functions

We start with the longitudinal (left) and transversal (right) spectra at the lowest available temperature $T=0.152$ GeV plotted in Fig. 7 for different spatial momenta. Lighter colors correspond to higher momenta. These reconstructions correspond to the most robust ones, since the underlying correlators are resolved with the largest number $N_{q_4}/2=10$ of imaginary frequencies in our ensemble. At the lowest momenta available, the spectrum exhibits a characteristic peak–trough structure. A well defined lowest lying positive peak is followed by a negative valley, which approaches the *x*-axis from below. The amplitude of both structures diminishes as one increases momenta. Consistently the trough in the transverse spectrum is more strongly pronounced than in the longitudinal sector. Their position is clearly correlated with spatial momentum, which we will study quantitatively in the next section.

Qualitatively the observed structures agree with expectations from continuum computations, which predict that the gluon spectrum at large frequencies will be negative and will asymptote to zero [40, 46]. Due to the ill-posed nature of the reconstruction task, the reconstructed spectra, however, show artificial oscillations around the *x*-axis with a monotonously decreasing amplitude, which both precludes both a sensible quantitative comparison to the asymptotic form and to the continuum zero-area sum rule. We note that close to the frequency origin the spectral function may start out flat or even with a small negative slope, related to a possible flattening off of the correlator close to $q_4=0$ [59].

Even though we cannot perform a systematic comparison of the reconstructions at different lattice spacing we plot the outcome of the corresponding low-temperature reconstructions in Fig. 8. We observe that the magnitude of the negative contributions appear to show a mild dependence on the lattice spacing. On coarser lattices, e.g. at $\beta =1.90$, the trough in the confined phase is less strongly pronounced than for $\beta =2.10$ (keeping in mind the slightly different temperatures and momenta). This behavior of a sharpening in the peak–trough structure is expected [23], since the continuum $T=0$ spectral function contains a pole at which the spectrum changes sign.

**Fig. 9:**
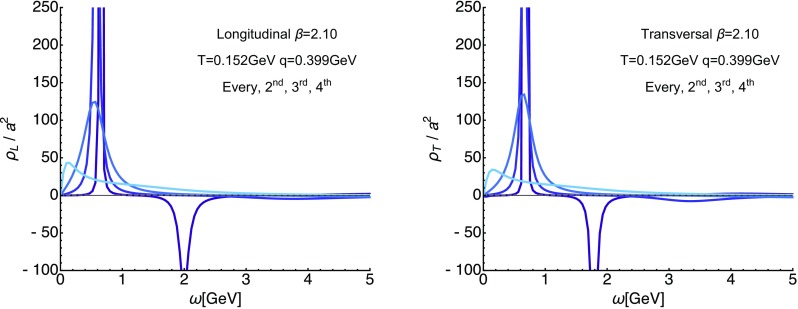
Systematic uncertainties of the spectral reconstruction in a fixed-scale approach for the longitudinal (left) and transversal (right) sector at $\beta =2.10$. We show the spectral functions extracted from the correlator at the smallest available $|\mathbf {q}|$ and lowest $T=0.152$ GeV, i.e. the dataset with the largest number $N_{q_4}/2=10$ of resolved imaginary frequencies (dark blue). The lighter blue solid curves denote the results from a sparsened dataset, using only every 2nd, 3rd or 4th imaginary frequency (dark to light colors). These thinned out correlators correspond to the reconstructed correlator [60] at higher temperature. Clear changes in the overall features of the reconstructed spectra are visible, in particular the negative trough is much more weakly resolved. The position of the first peak, however, remains relatively stable down to $N_{q_4}/2=4$, in particular in the transversal sector

**Fig. 10:**
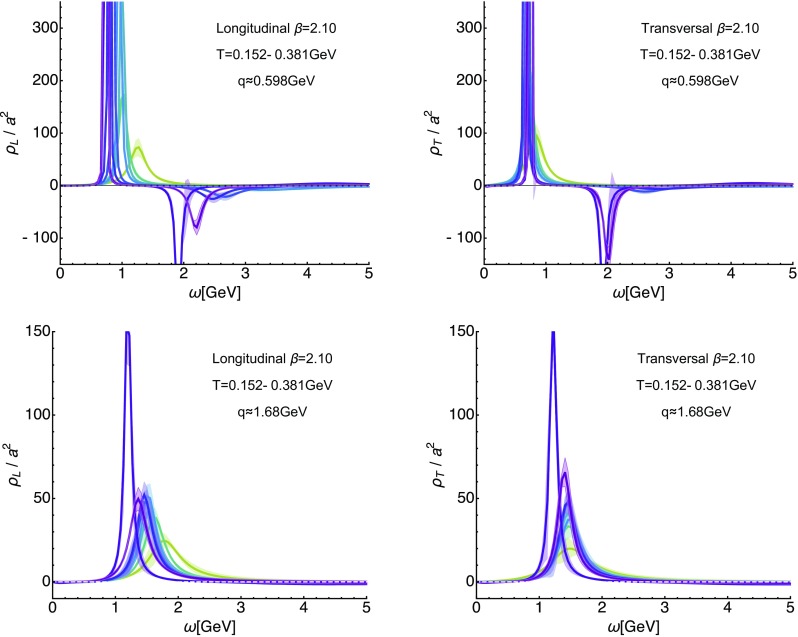
In-medium longitudinal (left) and transversal (right) gluon spectral functions at $\beta =2.10$ at the lowest available spatial momentum $q\approx 0.6$ GeV (top) and at intermediate $q\approx 1.68$ GeV (bottom) evaluated in the temperature range $T=0.152\ldots 0.381$ GeV

Before turning to the investigation of the temperature dependence of the gluon spectral function we need to consider another systematic error, which is present in the fixed-scale approach and related to the way how temperature is varied. The fact that at higher temperatures a smaller number of datapoints in imaginary frequencies are present, leads to a degradation of the spectral reconstruction. That is, when we find changes in the peak–trough structure at higher *T*, we need to make sure that what we observe is genuine in-medium physics and not simply a methods artifact.

**Fig. 11:**
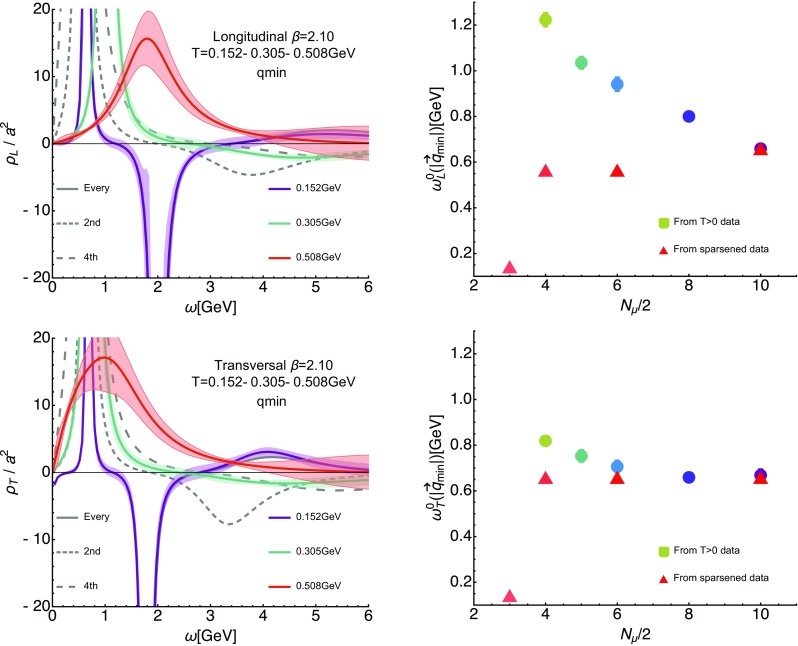
Comparison of the finite-temperature spectral reconstructions to those based on sparsened low-temperature longitudinal (top row) and transversal (bottom row) correlators at $\beta =2.10$. In the left column we show the reconstructions at the lowest $T=0.152$ GeV, an intermediate $T=0.305$ GeV and a high $T=0.508$ GeV as colored solid lines together with the reconstructions based on the sparsened data at $N_{q_4}=20,10,5$ as gray dashed lines. At $T=0.305$ GeV we find that the actual $T>0$ reconstruction (green) shows a clear sign that the trough has weakened more strongly than what a degradation in reconstruction resolution (gray small dashed line) alone results in. (Right) Comparison of the actual finite-temperature values of the dominant peak position $\omega _{L/T}^0(|\mathbf {q}_\mathrm{min}|)$ (circles) to the peak position reconstructed from the sparsened low-temperature correlator (red triangles). We see that the systematic error from the truncation pushes the values to lower values, while the tendency observed in the actual $T>0$ data is in the opposite direction

To this end we carry out the following crosscheck: We artificially reduce the input data (for a similar test in the context of non-relativistic spectral functions see [11]). At the lowest available temperature, i.e. the $\beta =2.10$ ensemble with $N_{q_4}=20$, we discard from the imaginary frequency correlator every 2nd, 3rd or 4th datapoint and feed these thinned out datasets into the reconstruction algorithm. This construction corresponds to carrying out the reconstruction on the *reconstructed correlator* (see also [60]), i.e. the correlator, which ensues if the low-temperature spectrum was present in a system at high temperatures.

In Fig. 9 we plot the reconstructed spectra based on the thinned out correlators at the smallest spatial momentum $|\mathbf {q}|=0.4$ GeV. The reconstructions are carried out for a single choice of default model $m=1,h=1$, since we are interested here simply in identifying possible systematic trends induced by the deterioration of the dataset. And as expected, the lower the number of input datapoints, the less structure the reconstructions contain, in particular the strength of the negative trough is strongly dependent on the $N_{q_4}$ used. On the other hand the position of the lowest lying peak is quite robust in the transversal sector and only for $N_{q_4}/2=3$ deviates strongly from all other reconstructions. In the longitudinal sector, reducing the underlying correlator data points already leads to a trend of shifting the peak position to lower frequencies when using half the original number.

Keeping these systematics in mind we can now proceed to an investigation of the in-medium modification of the gluon spectra.

### Temperature dependence of the spectral functions

We continue by inspecting the outcome of the spectral reconstructions, based on actual in-medium correlators, as shown in Fig. 10. The left panel contains longitudinal, the right panel the transversal ones. In the upper row reconstructions are obtained at the lowest available spatial momentum $q\approx 0.6$ GeV on the $\beta =2.10$ lattices, while in the bottom row we show the results for intermediate $q\approx 1.68$ GeV. The temperature range covered lies between $T=0.152\ldots 0.381$ GeV and the eight temperatures shown here correspond to those at which the default-model dependence was mild enough for a robust determination of the positive peak feature.

As can be expected from the discussion of the temperature dependence of the correlators, the longitudinal spectra show significantly stronger changes with increasing temperature than the transversal ones. At the same time the in-medium modification (in absolute terms) at low momenta appears as pronounced as at higher momenta. Qualitatively the changes are nonetheless similar in both the longitudinal and the transverse sectors. The lowest lying peak broadens, moves to higher frequencies and diminishes in height. Still at $T=0.381$ GeV we find clear indications for the presence of such a quasi-particle structure also in the longitudinal sector. For larger temperatures the statistical significance of the results is insufficient and we have refrained from presenting them. The observed behavior is consistent with an increase in the mass of a gluon quasi-particle and thus signals an increase in the strength of screening affecting the interactions mediated by it. At the same time the depth of the negative trough at low momenta appears to weaken. However, its width also seems to broaden and its minimum shifts to higher frequencies.

At higher momenta the trough is barely visible, even at the lowest temperature, as already indicated by Fig. 7. If one zooms in around the *x*-axis one would find that the systematic uncertainties make the result compatible with no negative trough present at all, even though the mean value of the reconstructed spectra always shows a regime where it falls below zero. We hence expect that with improved statistics and an increased number of datapoints $N_{q_4}$ the trough would eventually be recovered.

**Fig. 12:**
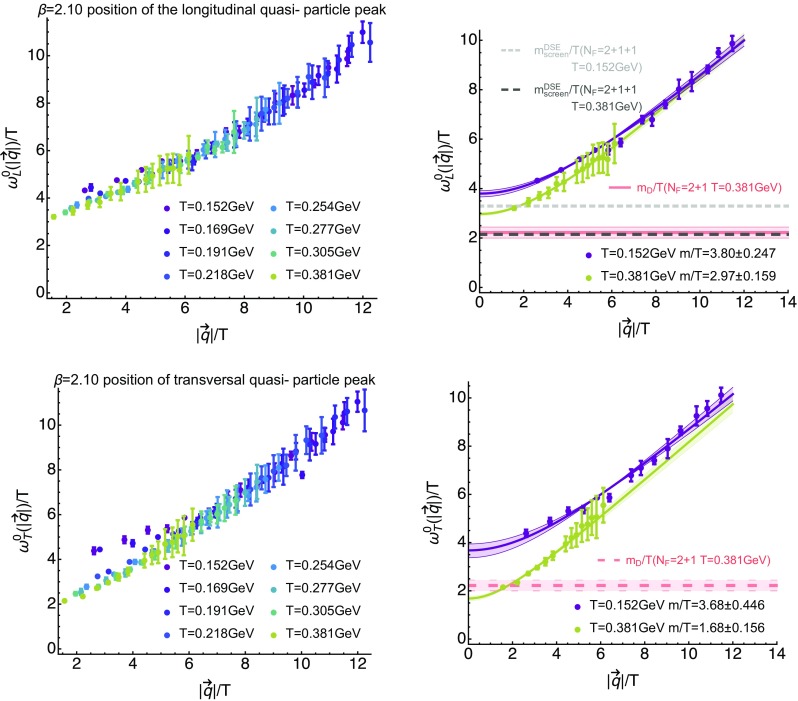
Momentum dependence of the longitudinal (top row) and transversal (bottom row) quasi-particle peak position at $\beta =2.10$. In the left column we show $\omega _{L/T}^0$ for those eight temperatures at which the default-model dependence was mild enough for a robust determination and we find that all of them take on a non-zero value for small spatial momenta. In both the longitudinal and the transversal sectors the reconstructions at the two lowest temperatures within the hadronic phase seem to exhibit a larger intercept than the curves in the deconfined phase. (Right) Fit of the lowest and highest temperature curves with the ansatz $\omega _L^0(|\mathbf {q}|)=A\sqrt{B^2+|\mathbf {q}|^2}$. The mass of the quasi-particle excitation is defined as the value of the intercept $m=AB$. As comparison to results in the literature we include here on the one hand the Debye mass evaluated from the heavy-quark potential in $N_f=2+1$ lattice QCD as red solid line. On the other hand we show for the longitudinal sector previous estimates of the electric screening mass obtained from Dyson–Schwinger computations including $N_f=2+1+1$ quark flavors as gray dashed lines

Our interest, however, lies mainly in determining the properties of the low lying positive peak, since e.g. its position can be interpreted as representing the in-medium dispersion relation $\omega ^0_{L/T}(|\mathbf {q}|)$ of a gluon quasi-particle, which in turn may become part of a dynamical model of the quark–gluon plasma. The peak width would inform us about the lifetime of such a quasi-particle excitation. However, due to the small number of available correlator datapoints, the observed values of the width are still dominated by reconstruction uncertainties.

Before embarking on a quantitative investigation of the quasi-particle peak, we need to ascertain whether the changes observed here are genuine in-medium effects. It is here that we can utilize the reconstructions based on the sparsened low-temperature datasets, as shown for the longitudinal (top row) and transversal (bottom row) sector in Fig. 11.

In the left column we contrast the actual in-medium reconstructions (solid colored lines) at $T=0.152$ GeV, $T=0.305$ GeV and $T=0.508$ GeV with those from sparsened low-temperature datasets at $N_{q_4}=20,10,5$ (gray dashed). What is important here is to observe that at $T=0.305$ GeV (green curve) the in-medium results show a weakened but nevertheless present negative trough. It is shallower than what can be explained simply by the degradation of the spectral reconstruction itself, visible in the gray short dashed curve. The $T=0.508$ GeV result already carries quite large error-bands, so that no significant difference regarding the negative trough compared to the sparsened data result can be found.

In the right column of Fig. 11 we now turn to the changes observed in the peak position, defined here naively via the topmost point of the reconstructed spectrum. We already saw that the effect of truncating the correlator is a shift to lower frequencies, while the in-medium spectra show a shift to higher frequencies. This difference is quantified here using the colored points, corresponding to the peak positions in the actual $T>0$ reconstructions, and the red triangles, which denote the position obtained in the sparsened reconstructions.

We conclude that both the observation of a diminishing trough depth and the shift of the positive peak position to higher frequencies with increasing temperature are genuine in-medium effects, which go significantly beyond the uncertainties introduced by the reduced number of datapoints.

### In-medium gluon dispersion relation

Having inspected the behavior of the reconstructed spectra qualitatively, we proceed to quantitatively determine position of the first positive peak $\omega _{L/T}^0$. In the left panel of Fig. 12 we show the peak positions, again defined naively via its topmost point, plotted against spatial momentum for those eight temperatures at which the default-model dependence was mild enough for a robust determination. Both the peak position and the momenta are rescaled by the temperature, which allows a straight-forward comparison of non-trivial differences in the behavior of $\omega _{L/T}^0$ at different temperatures. The errorbars arise from the variation of the results among changing both $m(\omega )$ and $h(\omega )$ as described in Sect. 2.2.

We find that all curves approach the *y*-axis at a non-zero value and that above $|\mathbf {q}|/T\approx 6$ GeV all of them exhibit an identical behavior within the relatively large systematic errorbars. As the number of correlator points reduces with increasing temperature, the size of the combined systematic and statistical errorbars increases concurrently. In turn we are not able to distinguish differences among the peak positions in the deconfined phase at low momenta. On the other hand in the confined phase, i.e. for the lowest two temperatures, the peak position seems to flatten off at a value above that in the deconfined phase. This difference is probed more quantitatively in the right panel of Fig. 12, where the peak positions for $T=0.152$ GeV$<T_c$ and $T=0.381$ GeV $>T_c$ are plotted together with a modified free-theory fit, $\omega _{L/T}^0(|\mathbf {q}|)=A\sqrt{B^2+|\mathbf {q}|^2}$. This simple fit ansatz manages to retrace the values reasonably well and leads to significantly different intercepts, i.e. quasi-particle masses, between the lowest and highest temperature both in the longitudinal sector,

32 $$\begin{aligned}&\left. m_L/T\right| _{T=0.152\,\mathrm{GeV}}=3.80\pm 0.25, \end{aligned}$$

33 $$\begin{aligned}&\left. m_L/T\right| _{T=0.381\,\mathrm{GeV}}=2.97\pm 0.16, \end{aligned}$$

and the transversal sector,

34 $$\begin{aligned}&\left. m_T/T\right| _{T=0.152\,\mathrm{GeV}}=3.68\pm 0.45 , \end{aligned}$$

35 $$\begin{aligned}&\left. m_T/T\right| _{T=0.381\,\mathrm{GeV}}=1.68\pm 0.16. \end{aligned}$$

The difference in the in-medium masses between the longitudinal and transversal sector at high temperature is qualitatively consistent with the expectations from weak coupling considerations. In a perturbative setting the strong coupling *g* is small and the electric scale of Debye screening $\sim g T$ is expected to be well separated from the non-perturbative magnetic sector, $\sim g^2 T$. The corresponding magnetic in-medium mass therefore will be smaller than its electric Debye counterpart.

For comparison purposes we also display (red solid curve) a recent lattice QCD determination of the Debye mass with $N_f=2+1$ flavors of light HISQ quarks [13, 61], which at $T=0.381$ GeV coincidentally agrees within errors with the HTL value of $m_D$ evaluated for four massless flavors at the scale $\mu =2\pi T$. Even at $T=0.381$ GeV though, the Debye mass result is consistently smaller than what is observed as quasi-particle mass directly from the gluon spectra. In addition we have included for the longitudinal sector previous estimates [33] for the electric screening mass computed in a Dyson–Schwinger approach including $N_f=2+1+1$ quark flavors. What we find is that at low temperatures their continuum values differ from our finite lattice spacing value by around 13%, with the functional result lying below the lattice one. At high temperatures the Dyson–Schwinger result agrees very well with the HTL Debye mass estimate and thus also lies below the gluon spectral function result.

## Conclusion

We have presented the first computation of finite-temperature gluon correlation and spectral functions in Landau gauge on full QCD ensembles with $N_f=2\,+1+1$ flavors of dynamical quarks, generated by the tmfT collaboration and gauge fixed using the cuLGT library on GPU’s. Based on these data we both carried out a Gribov–Stingl fit analysis of the correlators themselves and a Bayesian investigation of the corresponding gluon spectral functions. It is the first Bayesian study in this context, which is independent of the assumption of *O*(4) invariance, containing a systematic error budget. Spectral function reconstructions were performed with a novel Bayesian approach, which generalizes the recent BR method to arbitrary, i.e. non-positive-definite functions.

The outcome of the Gribov–Stingl fits is collected in Table 3 and the corresponding best fit curves plotted in Fig. 1. As was expected from perturbative computations at high temperature, the longitudinal correlators show a much stronger dependence on temperature than the transversal ones. We found that the temperature dependence of e.g. the quasi-particle mass parameter *r* in the fits shows a monotonous increase, which is qualitatively compatible with previous results obtained in quenched QCD. Since previous studies in $N_f=2$ full QCD used a fixed box approach and did not provide Gribov–Stingl fits on the renormalized propagators, no conclusive comparison could be made.

We further found (see Fig. 5) that, for a fixed momentum, at imaginary frequencies above the first Matsubara frequency $q_4\approx 2\pi T$ the correlator values lie already very close to their $T\approx 0$ behavior, while at $q_4=0$ significant differences between the correlators are manifest. At $q_4=0$ the correlator will however suffer most severely from the inherent finite extent of the Euclidean axis in standard lattice simulations. We have checked (see Fig. 6) that while interpolating the correlators using the assumption of *O*(4) scaling works reasonably well at small $q_4$, it degrades towards the boundaries of the Brillouin zone. The interpolation is found to work better on the longitudinal correlators than on the transversal ones but is globally not sufficiently precise to be deployed for the determination of spectral functions.

Our low-temperature reconstructed spectral functions (see Fig. 7) both in the longitudinal and transversal sector, show clear signs of positivity violation at high frequencies. In general we find one well defined positive peak structure at low frequencies, followed by a negative trough at higher $\omega$. At higher frequencies, the spectrum approaches the frequency axis from below, in qualitative agreement to the continuum asymptotic behavior. Due to the imperfections of the reconstruction process at high frequencies, the spectra however begin to artificially oscillate around the $\omega$-axis with a diminishing amplitude.

At higher temperatures (see Fig. 10) the spectral features change such that the positive peak structure broadens, moves to higher frequencies and shrinks, while the negative trough becomes more and more shallow and also moves to higher frequencies. In order to check whether these changes are actual in-medium effects, we carried out a systematic crosscheck based on reconstructions performed on sparsened low-temperature imaginary frequency datasets. It revealed (see Figs. 9 and 11) that the observed modifications of the peak and trough can indeed be attributed to in-medium physics. That is, while the overall form of the reconstructed spectra became more and more featureless, as the number of datapoints was reduced, the position of the lowest lying peak remained relatively stable down to $N_{q_4}/2=4$, moving slightly towards lower values. On the other hand the actual $T>0$ reconstructions showed a clear behavior of tending to larger values of $\omega ^0_{L/T}$.

Since a well pronounced positive peak at low frequencies was identified in all reconstructed spectra, we use its tip as a naive definition of a quasi-particle dispersion relation. The corresponding values plotted against spatial momentum (see Fig. 12) are compatible with a finite intercept at $|\mathbf {q}|=0$. Such an intercept, determined from a modified free-theory fit, can give a first rough estimate of the quasi-particle mass in the longitudinal and transversal sector (see (33) and (35)). We find that below $T_c$ the values of this mass appear to be consistent with each other for longitudinal and transversal gluons, while above the deconfinement transition a clear separation of values emerges. This difference is qualitatively consistent with the weak coupling expectation that the magnetic mass should be parametrically smaller than the Debye mass, which screens the electric fields. Comparing with previous results on QCD screening properties, either from the Debye mass extracted from the heavy-quark potential or a direct computation of the electric screening mass in a $N_f=2+1+1$ Dyson–Schwinger approach, shows values that are consistently smaller than our lattice estimates, the difference being around 13% at low temperatures and close to 28% at the highest temperatures investigated.

Our findings are encouraging: it appears to be possible to reconstruct characteristic features of gluon spectral functions from Landau-gauge lattice QCD correlators with a relatively small number of available frequency points, since the statistics of the ensembles is high. The position of the lowest lying peak structure is one example. To connect to the perturbative high momentum regime, where the signal to noise ratio in the correlators is still weak, will require increasing the statistics further. The quasi-particle peak width on the other hand demands simulations with a significantly larger number of temporal lattice points, i.e. a smaller lattice spacing. Connecting our lattice results on gluon spectra to e.g. the PHSD framework therefore needs to be postponed to future studies. Performing the full continuum extrapolation on the correlators and subsequent reconstructions has to be attempted in a future study as the current ensembles are not tuned for this purpose and thus feature a too coarse temperature resolution.

Already with the currently available data we may attempt to use the reconstructed spectra in a self-consistent computation for transport coefficients in full QCD, which is work in progress.

We are confident that with a further increase of the statistics on the tmfT ensembles and subsequently on the computed correlators the determination of the quasi-particle dispersion relation can be brought to a more robust quantitative level, in particular that it will become possible to resolve more clearly the temperature dependence of its intercept at $|\mathbf {q}|=0$.

One open problem left is to estimate the influence of the number of light-quark species and of the light-quark mass on the properties of the gluon spectral functions. Considering the ongoing work on thermodynamics for $N_f=2\,+1+1$ flavors, we will be able in the near future to investigate the case of more realistic light-quark masses (pion masses of close to 200 MeV) for $N_f=2\,+1+1$ flavors along the lines of the present paper. For a sensible comparison with $N_f=2$ simulations one would have to return to the previous tmfT datasets and carry out a careful reanalysis of the Gribov–Stingl fits taking into account the renormalization of the quasi-particle masses and widths.
